# Fungi Associated with Postharvest Diseases of Sweet Potato Storage Roots and In Vitro Antagonistic Assay of *Trichoderma* *harzianum* against the Diseases

**DOI:** 10.3390/jof7110927

**Published:** 2021-10-31

**Authors:** Narayan Chandra Paul, Soyoon Park, Haifeng Liu, Ju Gyeong Lee, Gui Hwan Han, Hyunsook Kim, Hyunkyu Sang

**Affiliations:** 1 Department of Integrative Food, Bioscience and Biotechnology, Chonnam National University, Gwangju 61186, Korea; ncpaulcnu@gmail.com (N.C.P.); psyangel0522@gmail.com (S.P.); liuhaifeng141@gmail.com (H.L.); wnrud2338@gmail.com (J.G.L.); 2 Kumho Life Science Laboratory, Chonnam National University, Gwangju 61186, Korea; 3 Center for Industrialization of Agricultural and Livestock Microorganisms, Jeongeup-si 56212, Korea; ghhan@cialm.or.kr; 4 Boran Pharma, Seoul 04206, Korea; kimhs@glovax.com

**Keywords:** fungal pathogens, postharvest disease, sweet potato, storage root, *Penicillium rotoruae*, *Aspergillus wentii*

## Abstract

Sweet potato is the 11th most important food crop in the world and an excellent source of nutrition. Postharvest diseases were monitored in sweet potato storage roots collected from the local markets in Korea during 2021. Several diseases including Fusarium surface and root rot, charcoal rot, dry rot, and soft rot were observed in the postharvest sweet potatoes. A total of 68 fungal isolates were obtained from the diseased samples, and the isolates were grouped into 8 different fungal colony types. Based on multilocus phylogeny and morphological analysis of 17 representative isolates, the isolates were identified as *Fusarium oxysporum*, *F. ipomoeae*, *F. solani*, *Penicillium citrinum*, *P.* *rotoruae*, *Aspergillus* *wentii*, *Mucor* *variicolumellatus* (*Mu. circinelloides* species complex), and *Macrophomina phaseolina*. *F. oxysporum* was the predominant pathogen as this is the most common pathogen of sweet potato storage roots causing the surface rot disease, and *M. phaseolina* caused the most severe disease among the pathogens. Dual culture antagonistic assays were evaluated using *Trichoderma harzianum* strains CMML20–26 and CMML20–27. The results revealed that the two strains showed strong antifungal activity in different ranges against all tested pathogens. This study provides an understanding of diverse postharvest diseases in sweet potatoes and suggests potential biocontrol agents to manage the diseases. In addition, this is the first report of sweet potato storage root rot diseases caused by *A. wentii*, and *P. rotoruae* worldwide.

## 1. Introduction

The sweet potato (*Ipomoea batatas* (L.) Lam.; Convolvulaceae) is regarded as one of the most important food crops in the world and is an alternative source of bioenergy, with an annual production area of 8.0 million hectares and a total global production of 106,569 million tons [1,2]. Its storage roots and leaves can be used as a staple food, animal feed, and supplementary food such as chips and starch production [3,4]. Therefore, this crop is now regarded as a high-priority crop targeted for reducing food insecurity and malnutrition in many countries [5]. Furthermore, sweet potato is an excellent source of nutrients, including vitamins, potassium, iron, calcium, and minerals with medicinal value owing to its anti-cancer, anti-diabetic, and anti-inflammatory activities [6,7,8,9,10]. Additionally, functional food products, such as β-carotene and anthocyanins, come from sweet potato, making it a source of novel natural health-promoting compounds [8].

There are several biotic and abiotic factors limiting the production and commercialization of sweet potatoes. Among them, fungal diseases are the most prominent before and after harvest; fungi reduce the quality of storage roots [11,12]. The periderm of the sweet potato is thinner, increasing the risk of storage diseases and causing severe losses during storage [12,13]. Moreover, the presence of postharvest pathogens affects both the appearance and taste of the products [14]. Commonly observed postharvest diseases caused by fungi include black rot (*Ceratocystis fimbriata*), dry rot (*A. niger* and *Diaporthe batatas*), Fusarium surface rot (*F. oxysporum*), Fusarium root and end rot (*F. solani*), foot rot (*Plenodomus destruens*), soft rot (*Rhizopus stolonifer* and *Rhizopus oryzae*), blue mold (*Penicillium* spp.), java black rot (*Botryodiplodia theobromae*), circular spot (*Sclerotium rolfsii*), charcoal rot (*M. phaseolina*), and storage rot (*Mucor* sp.) [3,5,6,10,13,14,15,16,17,18]. Some pathogens such as *Fusarium* spp. and *M. phaseolina* can survive in crop residue and soil from one season to another and enter the storage roots through wounding, causing diseases postharvest [5]. Other high decay losses result from tip rot, which is characterized by visible decay at one or both ends of the storage roots. The types of pathogens involved in tip decay have been inconsistent; common pathogens isolated include *F. solani*, *M. phaseolina*, *B. theobromae*, and *D. batatas* [5,19,20].

Cultural practices are generally applied to overcome or minimize the problems associated with the storage of sweet potatoes. In addition, chemicals can also be applied to remove pathogens from storage roots [21,22]. However, it is believed that chemical applications threaten the quality and safety of food. In agriculture, farmers rely heavily on synthetic fungicides or pesticides to successfully control plant diseases. However, the environmental pollution caused by excessive use of agrochemicals is of worldwide concern. Therefore, development of alternative methods is actively being researched, such as microbially sourced antifungal agents and chemicals [21,23].

In the present study, sweet potato samples were collected from the local markets of three locations in Korea, and different symptoms of postharvest diseases were observed. Therefore, this study aimed (i) to investigate the postharvest fungal diseases of sweet potato in Korea and characterize the causal fungal isolates by molecular phylogenetic and morphological analyses and pathogenicity assay, and (ii) to test in vitro biocontrol activity against the fungal pathogens by two *T. harzianum* strains.

## 2. Materials and Methods

### 2.1. Fungal Isolation

**Figure 1:**
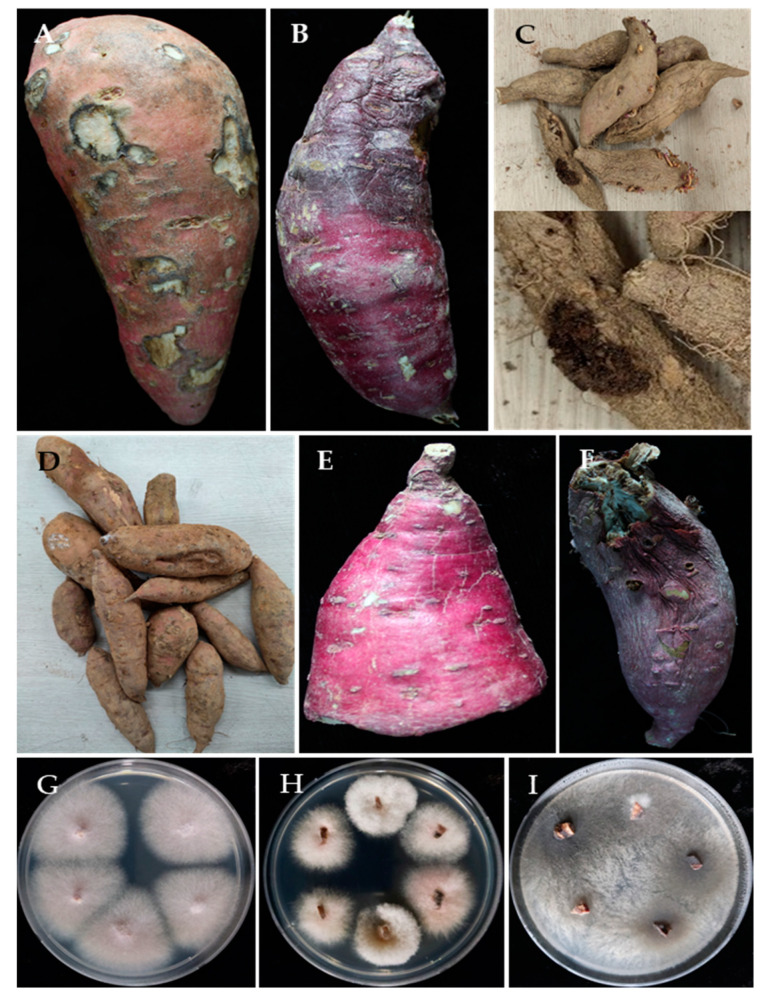
Symptoms of sweet potato postharvest diseases collected from local markets in Korea: (**A**) Fusarium surface rot, (**B**) charcoal rot, (**C**) *Aspergillus* mold, (**D**) surface rot, (**E**) end rot, and (**F**) *Penicillium* mold. The surface sterilized storage root tissues of Fusarium rot, charcoal rot, and other diseases were placed on potato dextrose agar media containing antibiotics. Examples of fungal colonies grown from the tissues of Fusarium rot (**G**,**H**) and charcoal rot (**I**) are shown.

Sweet potato storage root samples were collected from the market of three different locations in Korea in 2021, and the locations were Buan-gun, Cheonan-si, and Haenam-gun. Samples were kept in polyethylene bags, brought to the laboratory, and stored in a refrigerator prior to isolation of pathogens. For the isolation, diseased storage roots were surface sterilized with 1% NaOCl solution for 5 min, washed three times with sterilized distilled water, and then air-dried on filter paper in a laminar airflow chamber. The storage roots were placed onto potato dextrose agar (PDA) supplemented with 50 μg mL^−1^ of streptomycin, rifampicin, and kanamycin (MB cell, Seoul, Korea) to stop bacterial growth. After incubation at 25 °C for 3–10 days, individual hyphal tips of the developing fungal colonies were placed onto PDA and further incubated for 5–10 days for culture purity. Finally, representative isolates were selected, assigned an identification number (CMML21–1 to CMML21–17), and preserved in the Molecular Microbiology Lab, Dept. of Integrative Food, Bioscience and Biotechnology, Chonnam National University, Gwangju, Republic of Korea (Table S1). The fungal isolates were preserved in 20% glycerol stock solution at −80 °C. The photographs of symptomatic sweet potato postharvest diseases and fungal colonies grown from the surface sterilized storage roots are shown in Figure 1.

### 2.2. DNA Extraction, PCR Amplification, and Sequencing

To confirm the identity of the fungi, total genomic DNA was extracted directly from the mycelia grown on PDA using the CTAB DNA-extraction method [24]. Different gene regions, the internal transcribed spacer (ITS), elongation factor 1-alpha (*EF1–α*), calmodulin (CAL), RNA polymerase II second largest subunit (RPB2), small subunit (SSU, large subunit (LSU), and β-tubulin (BT) genes were amplified using the primer pairs ITS1–ITS4, EF1 728F–EF1 986R, CAL1–CAL2, RPB2 5F–RPB2 7cR, NS1–NS4, LROR–LR5, and B27a–B27b, respectively [25,26]. PCR primers were chosen based on fungal genera. For the identification of *Fusarium* spp. and *Macrophomina* spp., primers of ITS and *EF1-α* regions were commonly used. Primers of ITS, SSU, and LSU regions were chosen for the identification of *Mucor* spp. To identify species level of *Aspergillus* and *Penicillium*, primer sets from ITS, BT, CAL, and RPB2 were used. The polymerase chain reaction (PCR) was carried out using a SimpliAmp™ PCR system by Applied biosystems^®^ (Thermofisher Scientific, MA, USA) in a 25 μL reaction volume containing 0.25 μL of Takara Ex Taq^®^ DNA polymerase (TaKaRa Bio Inc., Shiga, Japan) (5U μL^−1^), 2.5 μL of 10× Ex Taq buffer, 2 μL of dNTP mixture (2.5 mM each), 1 μL of each primer (10 pmoles μL^−1^), 1 μL template DNA solution (100 ng μL^−1^), and sterilized distilled water up to 25 μL. The PCR amplification conditions were initial denaturation at 98 °C for 30 s, followed by 35 cycles of denaturation at 98 °C for 15 s, and annealing at 50 °C for ITS, SSU and LSU, 55 °C for *EF1–α* and CAL, 49 °C for RPB2, 52 °C for BT, with 30 s, and a final extension at 72 °C for 2 min. PCR products were sequenced in both directions by a commercial sequencing service provider (Macrogen, Daejeon, Korea).

### 2.3. Molecular Phylogeny

Sequences were manually adjusted with a MEGA X program [27] and subjected to Basic Local Alignment Search Tool (BLASTN) searches using the National Center for Biotechnology Information (NCBI) database (http://www.ncbi.nlm.nih.gov) to obtain sequence similarity. Closely related sequences were obtained from GenBank (Table S2) for phylogenetic analysis, adjusted manually using the MEGA X program [27], and aligned using ClustalX v1.83 [28]. The sequence ends were trimmed manually to remove low–quality bases using the BioEdit v5.0.9.1 program [29]. Maximum likelihood (ML) analysis was performed using the MEGA X program [27] to construct phylogenetic trees.

### 2.4. Morphology

To determine colony morphology, representative isolates were cultured on different media depending on the fungal genera, including potato dextrose agar (PDA), malt extract agar (MEA), Czapek yeast extract agar (CYA), and yeast extract sucrose agar (YES) at 25 °C in the dark for 7 days. To microscopically observe the conidia, mycelia of the isolates were scratched off after 7 days and incubated under NUV (near–ultraviolet) light in 12h–12h light–dark conditions for 3–5 days [30]. The size and shape of the conidia, conidiophores and other morphological characteristics were measured using a microscope (Olympus, Tokyo, Japan). Color names were assigned using ‘A Morphological Colour Chart’ [31]. Morphological characteristics of the isolates were then compared with previous descriptions.

### 2.5. Pathogenicity and Reisolation

Pathogenicity assay was performed on a susceptible sweet potato variety, namely Beniharuka, with the isolates CMML21–2, CMML21–4, CMML21–5, CMML21–7, CMML21–8, CMML21–12, CMML21–13, CMML21–16, and CMML21–17. Each isolate was selected from each species group, and two of the isolates (CMML21–4 and CMML21–7) had not previously been identified in sweet potatoes anywhere in the world. Beniharuka is known to be susceptible to common sweet potato diseases. To perform the pathogenicity test, the fungal isolates were cultured on PDA for 7–10 days, and PDA plugs were used to inoculate sweet potato storage roots. The sweet potato storage roots were surface sterilized by dipping in 1% NaOCl for 10 min and washed with sterilized distilled water three times. The storage roots were then allowed to dry in a laminar airflow chamber. A 5 mm hyphal disc of each isolate was placed in a wound made in each root using a 5 mm cork borer. Blank PDA discs were used as control treatments [15]. Additionally, the spore suspension method was applied for the newly detected sweet potato pathogens. Sporulation was observed after culturing in PDA for 5–7 days; spores were collected and counted with a hemocytometer. Spore suspensions (20 µL) of the *Penicillium* and *Aspergillus* isolates were inoculated following the methods described by Paul et al. [10]. Spore suspensions were adjusted to 1 × 10^5^ spores mL^−1^ before inoculation. Identical amounts of sterilized distilled water served as the control. The storage roots were then kept in moistened clean boxes and incubated at 25 °C. The pathogenicity test was conducted at three different times with three replications each time. After one week of incubation, the artificially inoculated storage roots were observed for lesion development daily; after 3 weeks, pathogenicity was confirmed, and disease severity was measured. Diseased storage roots were used for the reisolation of the fungi on PDA media. After reisolating, the fungal morphology was observed to confirm Koch’s postulates.

### 2.6. In Vitro Biocontrol Activity

Pathogens causing postharvest diseases of sweet potatoes were screened for antifungal activity with two antagonistic *Trichoderma harzianum* strains (CMML20–26 and CMML20–27) collected from the Molecular Microbiology Laboratory, Dept. of Integrative Food Bioscience and Biotechnology, Chonnam National University, Gwangju, Republic of Korea. Nine representative pathogenic species were selected for screening: *F. oxysporum* CMML21–2, *A. wentii* CMML21–4, *P. expansum* CMML21–5, *P. rotoruae* CMML21–7, *F. ipomoeae* CMML21–8, *Mu. variicolumellatus* CMML21–12, *F. oxysporum* CMML21–13, *M. phaseolina* CMML21–16, and *F. solani* CMML21–17. Five to seven days old antagonistic *T. harzianum* strains and pathogenic fungi were cultured on opposite edges of the PDA and incubated at 25 °C for 5–7 days. Each experiment was repeated three times. Antifungal activity was assessed by the size (diameter in mm) of the inhibition zones by following the formula explained by Ji et al. [23].

$$\mathrm{Inhibition}\;\mathrm{percentage}\;\left(\%\right)=\frac{A1-A2}{A1}\times 100$$

where, *A*1 = radial growth of pathogenic mycelia without the *T. harzianum* strain (CMML20–26 or CMML20–27), *A*2 = radial growth of pathogenic mycelia with the *T. harzianum* strain.

## 3. Results

### 3.1. Associated Pathogenic Fungi

Symptoms of different diseases were observed on the collected storage roots; surface rot disease was most common, followed by several molds. Other diseases found on the storage roots included dry rot, charcoal rot, and end rot. Surface rot symptom was characterized by its circular, somewhat sunken enlarged spots on the surface of the storage roots. Charcoal rot disease was restricted to part of sweet potato so that one end desiccated, and the other end appeared intact. The symptoms of charcoal rot and end rot were very similar, and two different pathogens were obtained from these symptoms. In blue mold symptom, the bluish-green sporulation was abundantly visible on the surface of the storage roots. Dry rot symptom was also found on the surface of the storage roots and became dried (Figure 1A–F). Sweet potato surface sterilized storage root tissues were plated on PDA to isolate the causal pathogens.

In the present study, a total of 68 fungal isolates were recovered; among them, 23, 25, and 20 isolates were from storage roots originating from Cheonan-si, Haenam-gun, and Buan-gun regions, respectively. The fungal colonies were compared and a total of 17 representatives were selected for further study based on colony morphology and colony characteristics. Colonies assumed to be *Fusarium* spp. were the most predominate fungi (67.6%) recovered in this experiment, followed by *Macrophomina* sp. (14.7%), *Penicillium* spp. (10.3%), *Aspergillus* sp. (5.9%), and *Mucor* sp. (1.5%) (Table 1 and Figure 1).

### 3.2. Molecular Phylogeny

**Figure 2:**
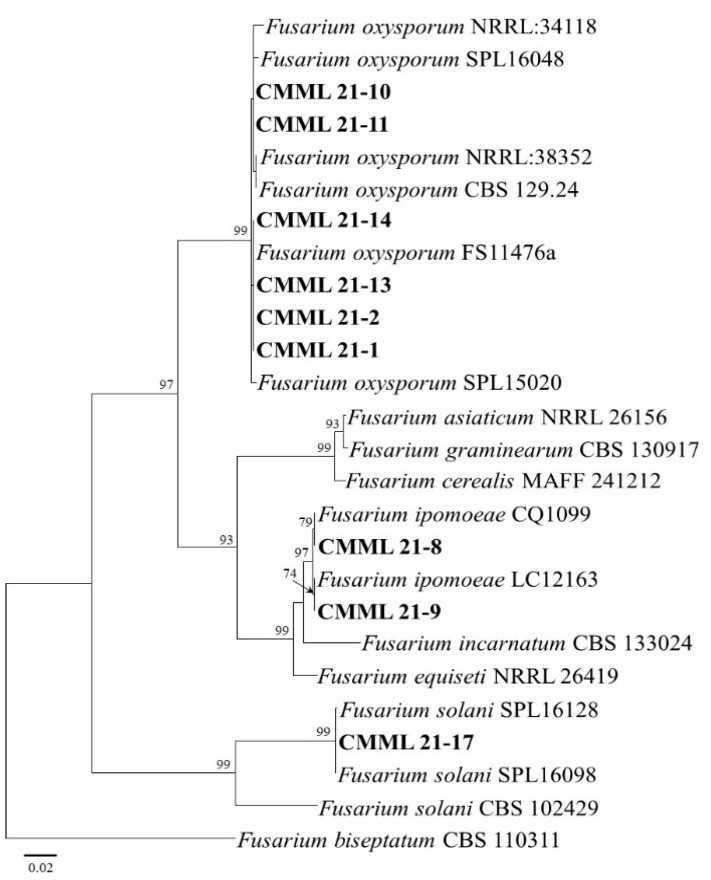
Maximum likelihood tree of the *Fusarium* isolates (CMML21–1, CMML21–2, CMML21–8, CMML21–9, CMML21–10, CMML21–11, CMML21–13, CMML21–14, and CMML21–17) inferred from the combined data sets of the ITS and *EF1–α* gene sequences constructed by the MEGA X program. The tree is rooted to *F. biseptatum* CBS 110311. Numbers on the branches indicate the bootstrap values. The scale bar indicates expected changes per site. The isolates from the present study are indicated in bold.

Based on BLASTN search analysis and molecular phylogeny, the isolates CMML21–1, CMML21–2, CMML21–10, CMML21–11, CMML21–13, and CMML21–14 were identified as *F. oxysporum*. Two sequences (ITS and *EF1–α*) of these isolates showed 99–100% sequence similarity with the reference strains (SPL 15020 and SPL 16048), which had previously been reported to be *F. oxysporum* in sweet potato in Korea [3]. The maximum likelihood phylogenetic tree showed that the six isolates and the reference *F. oxysporum* strains (CBS 129.24, FS11476a, NRRL:34118, NRRL:38352, SPL 15020, and SPL 16048) were grouped together, with a high bootstrap value (99%). In addition, isolates CMML21–8 and CMML21–9 were identified as *F. ipomoeae*, supported by high sequence similarity with the reference *F. ipomoeae* strains, and they formed a monophyletic group comprising the two isolates and *F. ipomoeae* strains (CQ1099 and LC12163), with a bootstrap value of 97%. A BLASTN search of ITS and *EF1–α* sequences from isolate CMML21–17 indicated that the isolate had high sequence similarity (99–100%) with *F. solani* reference strains, and the isolate was grouped with three *F. solani* strains with a high bootstrap value (%) (Figure 2).

**Figure 3:**
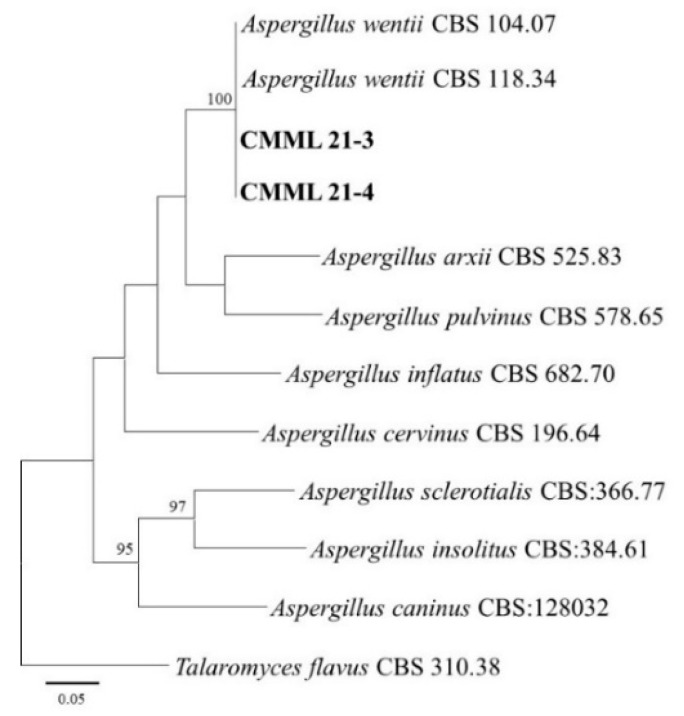
Maximum likelihood tree of the *Aspergillus* isolates (CMML21–3 and CMML21–4) inferred from the combined data sets of the ITS, BT, and CAL gene sequences constructed by the MEGA X program. The tree is rooted to *Talaromyces flavus* CBS 310.38. Numbers on the branches indicate the bootstrap values. The scale bar indicates expected changes per site. The isolates from the present study are indicated in bold.

For the identification of two isolates (CMML21–3 and CMML21–4) of *Aspergillus* species, three genes, including ITS, BT, and CAL, of isolates were sequenced. BLASTN search analysis and maximum likelihood phylogenetic analysis using three gene sequences (ITS+BT+CAL) revealed that the two isolates matched well with the reference strains (CBS 118.34 and CBS104.07) of *A. wentii* and formed a single clade in the phylogenetic tree with a high bootstrap value (100%) (Figure 3).

**Figure 4:**
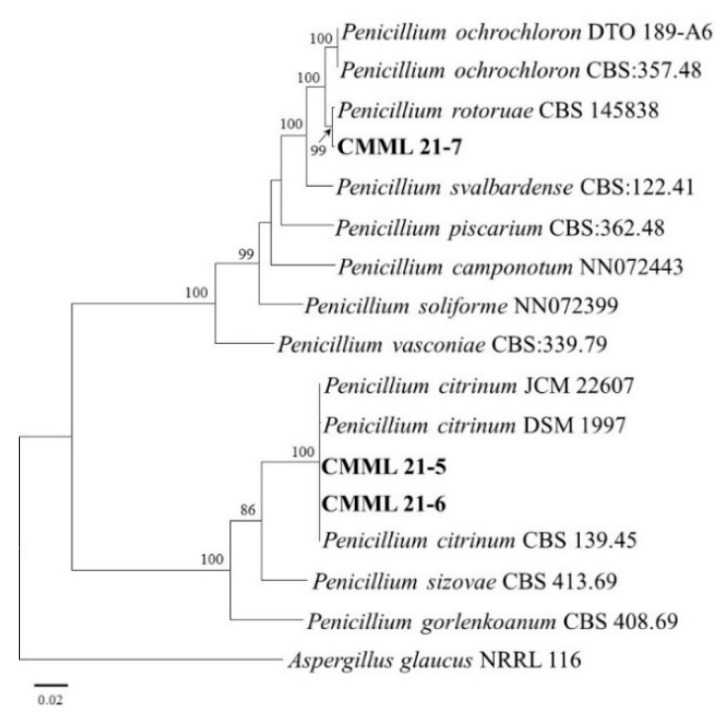
Maximum likelihood tree of the *Penicillium* isolates (CMML21–5, CMML21–6, and CMML21–7) constructed from the combined data sets of the ITS, RPB2, CAL, and BT gene sequences constructed by the MEGA X program. The tree is rooted to *Aspergillus glaucus* NRRL 116. Numbers on the branches indicate the bootstrap values. The scale bar indicates expected changes per site. The isolates from the present study are indicated in bold.

Two morphologically distinct *Penicillium* species causing mold disease in sweet potatoes were recovered. Isolates CMML21–5 and CMML21–6 were identified as *P. citrinum* based on a BLASTN search and molecular phylogenetic analyses using multigene sequences (ITS, RPB2, CAL, and BT). The two isolates and three reference *P. citrinum* strains (CBS 139.45, JCM 22607, and DSM 1997) were grouped with a high bootstrap value (100%). In addition, isolate CMML21–7 was identified as *P. rotoruae*, supported by the high sequence similarity with the *P. rotoruae* strain CBS 14.534 and forming a monophyletic group comprising the isolate and *P. rotoruae* strain, with a bootstrap value of 99% (Figure 4).

**Figure 5:**
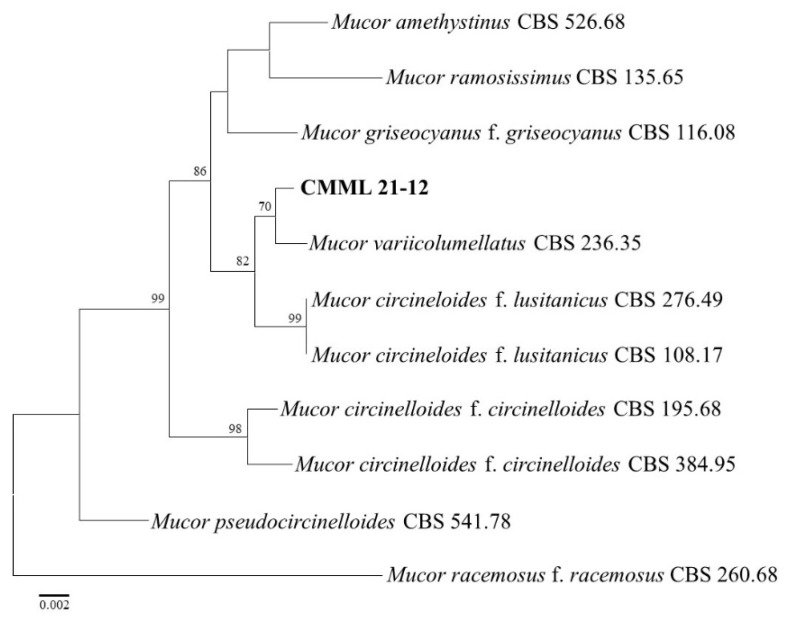
The maximum likelihood tree of the *Mucor* isolates (CMML21–12) was inferred from the combined datasets of the ITS, LSU, and SSU gene sequences constructed by the MEGA X program. The tree is rooted to *Mu. racemosus* f. *racemosus* CBS 260.68. The number on branches indicates the bootstrap values. The scale bar indicates expected changes per site. The isolates from the present study are indicated in bold.

**Figure 6:**
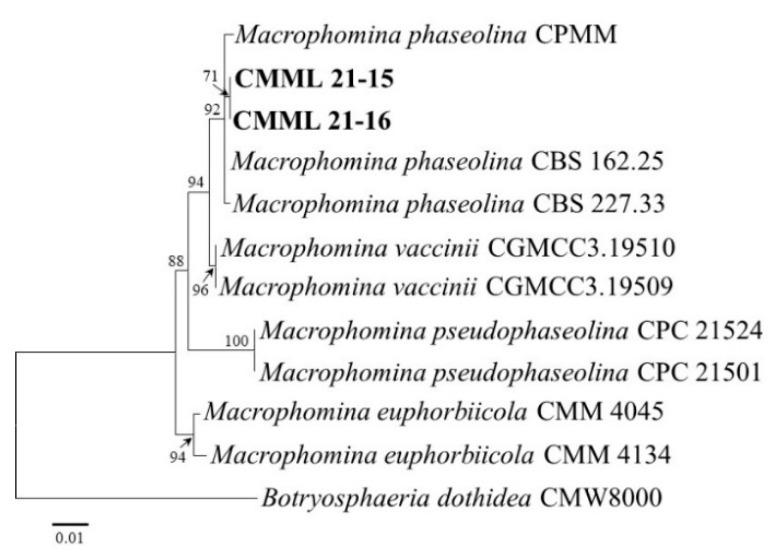
Maximum likelihood tree of the *Macrophomina* isolates (CMML21–15 and CMML21–16) inferred from the combined data sets of the ITS and *EF1–α* gene sequences constructed by the MEGA X program. The tree is rooted to *Botryosphaeria dothidea* CMW8000. Numbers on the branches indicate the bootstrap values. The scale bar indicates expected changes per site. The isolates from the present study are indicated in bold.

The isolate CMML21–12 obtained from soft rot-related disease in sweet potato was identified as *Mu. variicolumellatus* (*Mu. circinelloides* complex). Molecular phylogeny and the maximum likelihood tree constructed from the ITS, SSU, and LSU sequences showed that the isolate matched well with the reference strain of *Mu. variicolumellatus* (CBS 236.35) and was closely related to *Mu. circinelloides* f. *lusitanicus* (CBS 108.17 and CBS 276.49) (Figure 5). The causal agent of charcoal rot was isolated in this study. Two representative isolates (CMML21–15 and CMML21–16) were sequenced with ITS and *EF1–α;* the sequence and phylogenetic analyses revealed that the isolates were identified as *M. phaseolina* and matched well with the reference strains of *M. phaseolina* (CBS 277.33, CBS 162.25, and CPMM) (Figure 6).

### 3.3. Morphology

**Figure 7:**
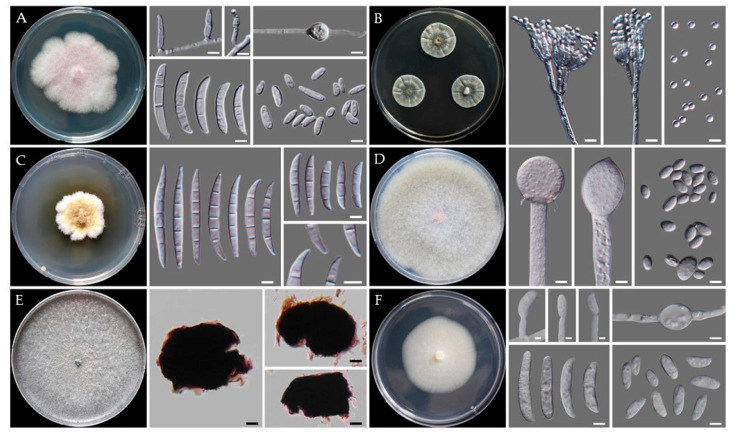
Morphological characteristics of representative isolates cultured on PDA or CYA for 7 days at 25 °C. (**A**) *F. oxysporum* CMML21–2, colony on PDA, monophialides, chlamydospores, macro, and microconidia; (**B**) *P. citrinum* CMML21–5, colony on CYA, conidiophores and conidia; (**C**) *F. ipomoeae* CMML21–8, colony on PDA, macroconidia, and typical structures of conidial ends; (**D**) *Mu.* *variicolumellatus* CMML21–12, colony on PDA, sporangium, and multi sized spores; (**E**) *M. phaseolina* CMML21–15, colony on PDA with sclerotia; and (**F**) *F. solani* CMML21–17, a colony on PDA, monophialides, intercalary chlamydospores, macro and microconidia. Scale bars, (**A**–**D**,**F**) = 5 μm and (**E**) = 10 μm.

For morphological analysis, fungal isolates were grown on different media (mostly PDA and MEA) at 25 °C in darkness to facilitate colony morphology, texture, color, and the sizes and shapes of conidia and conidiophores. Representative isolates from *Fusarium* species to characterize morphologically were CMML21–2, CMML21–8, and CMML21–17. The size of the macro and microconidia of the isolate CMML21–2 ranged from 14.5–33.0 × 2.7–4.4 µm to 5.6–10.2 × 2.5–3.8 µm (Figure 7A). The macroconidia were slightly curved or straight, usually with three septations, and the microconidia were elliptical to cylindrical with no septation. Morphologically, the isolate CMML21–2 was identical to the previous descriptions of *F. oxysporum* [3].

Morphology study of the isolate CMML21–8 revealed that the colony on PDA after 7 days at 25 °C was white, fluffy, and grew moderately quickly; cotton-like aerial mycelia were common. Conidia formation was hard; conidia were slightly curved and tapered at the apex. Conidial size varied and ranged between 17.5–49.0 × 3.1–5.6 to 5.1–8.0 × 3.1–4.7 µm, and 3–5 septations was common (Figure 7C). Microconidia were ovoid, single-celled, and non-septate, and the shapes were globose. Morphologically, the isolate was identical to the previously described *F. ipomoeae* [32,33,34,35]. The isolate CMML21–17 was grown on PDA for 7 days at 25 °C; the fungus produced sparse aerial mycelia, orange pigment on the agar, and whitish to off-white colonies. The macroconidia shape was cylindrical to falcate with rounded apical cells. The average length and width of the macroconidia were 10.5–30.6 × 4.1–5.7 μm, respectively. The septation of the conidial cells observed were one to four. The shape of the microconidia was fusiform to ovoid and varied in size with no or a rare single septation (Figure 7F). Based on the morphological and cultural characteristics, the fungal isolate was identified as *F. solani* [15].

The isolates CMML21–5 and CMML21–6 were identified as *P. citrinum*. CMML21–5 was cultured on MEA, CYA, and YES at 25 °C for 7 days. The reverse colors on CYA and YES were brownish-yellow and yellow or orange yellow, respectively. The size of the colonies varied with the media; the diameters on MEA, CYA, and YES were 34–40, 25–28, and 23–26 mm, respectively. Conidia were globose to sub-globose with smooth walls (Figure 7B). Moderate sporulation on CYA with bluish gray green conidia. Moderate to good sporulation on YES and strong, soluble yellow pigment were produced. On MEA, strong blue, velvety with small pale yellow exudate droplets were produced. Conidiophores biverticillate or terverticillate and abundant on fresh isolates with smooth-walled stipes; metulae in whorls, ranging from 10.0–14.0 × 2.2–2.8 µm in diameter; phialides ampuliform with sizes ranging from 6.4–9.1 × 1.6–2.2 µm. The isolates differed from relatives with yellow reverse on CYA, globose and smooth-walled conidia; sizes were 2.3–3.1 × 2.3–3.4 µm. Morphological features matched well with previous explanations of *P. citrinum* [36].

The isolate CMML21–12 was identified by molecular methods as *Mu. variicolumellatus*, which had previously been found in sweet potatoes [18]. The sporangia formed on repeatedly sympodially branched sporangiospores and were mostly two types of sporangiophores: short, intensive, sympodially branched and tall, less branched. The spores termed as sporangiospores were either subglobose and less than 15 (11.1–14.6) µm diameter or ellipsoidaland and less than 10 (4.2–9.1) µm diameter (Figure 7D). Based on its morphological characteristics, the species was identified as *Mu. variicolumellatus* [37,38].

Two isolates, CMML21–15 and CMML21–16, were isolated from the charcoal rot of the sweet potato. Morphologically, they were initially white but later became dark gray to blackish with many black, oblong microsclerotia on the PDA after culturing for 7 days at 25 °C. Aggregation of hyphae formed jet black microsclerotia, 26.7–90.1 × 26.3–52.2 µm in size. The microsclerotia were irregular in shape—some were round to oblong and smooth-walled (Figure 7E). These isolates were confirmed as *M. phaseolina* based on these explained characteristics [39,40,41].

### 3.4. Taxonomy: Aspergillus wentii CMML21–4

Isolates examined: CMML21–3 and CMML21–4.

Phylogeny: Three markers were used to identify the fungal species: ITS = OK104044–45, BT = OK104452–53, and CAL = OK104457–58. One more marker was used for sequencing: RPB2 = OK104462–63. Sequence analysis and a phylogenetic tree revealed that the isolates CMML21–3 and CMML21–4 completely matched with *A. wentii* CBS 104.04 and CBS 118.34.

**Figure 8:**
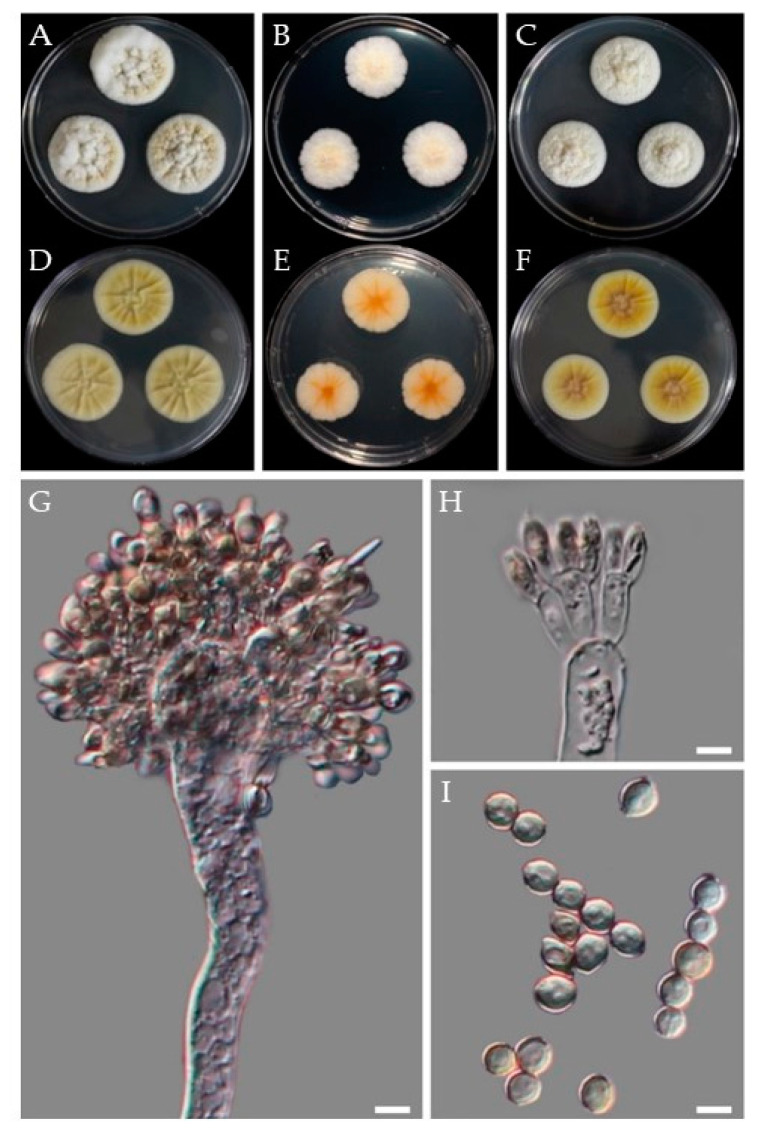
Morphological features of the isolate CMML21–4. Obverse and reverse colony morphology on CYA (**A**,**D**), MEA (**B**,**E**), and YES (**C**,**F**) after 7 days at 25 °C. Structures and shapes of conidiophores (**G**,**H**); conidia (**I**). Scale bars, (**G**–**I**) = 5 μm.

Morphology: On CYA at 25 °C: After 7 days colony diameter was 32–35 mm. Colonies white yellowish; exudates present; reverse ivory yellow to cream. Colonies radially furrowed, floccose (Figure 8A,D). On MEA at 25 °C, colonies white to yellow and reaching 23–26 mm in diameter, reverse light buff to light ochraceous salmon. Colony furrowed and floccose; conidia were splitting into loose narrow columns (Figure 8B,E). On YES at 25 °C: Colonies white to white to yellow; furrowed, reverse yellowish and reaching 28–30 mm in diameter. Exudate present (Figure 8C,F).

Conidiophore’s stipes smooth, hyaline to pale yellow brown; vesicles subglobose to globose or obovoid, ellipsoidal, 17.0–29.4 μm wide. Aspergilla biseriate, metulae covering 4/5 of the whole surface of the vesicle, 4.8–24.3 × 3.2–7.9 μm; phialides 6.7–8.5 × 2.6–4.0 μm. Conidia varied in shape and size; globose to ellipsoidal or doliiform, and 3.7–6.9 μm in size (Figure 8G–I).

Distribution: *A. wentii* is found in many crops, including cotton seeds, barley, rice, olives, pineapples, oats, nuts, pecans, groundnuts, wheat, and fir. The fungi are not limited to plants and vegetables but have also been associated with indoor mold and rhizospheric soils. This is the first worldwide report of *A. wentii*-caused disease of postharvest sweet potato storage roots.

Note: This fungus has been found in many countries, including China, the USA, Japan, and India but here for the first time in Korea.

### 3.5. Taxonomy: Penicillium rotoruae CMML21–7

Phylogeny: Four markers were used to identify the fungal species: ITS = OK104048, BT = OK104456, RBP2 = OK104466, and CAL = OK104461. Sequence analysis revealed that the isolate CMML21–7 matched completely with *P. rotoruae* CBS 145834. Relatives of the isolate are *P. ochrochloron* DTO 189–A6, CBS 357.48, and *P. svalbardense* CBS 122.41.

**Figure 9:**
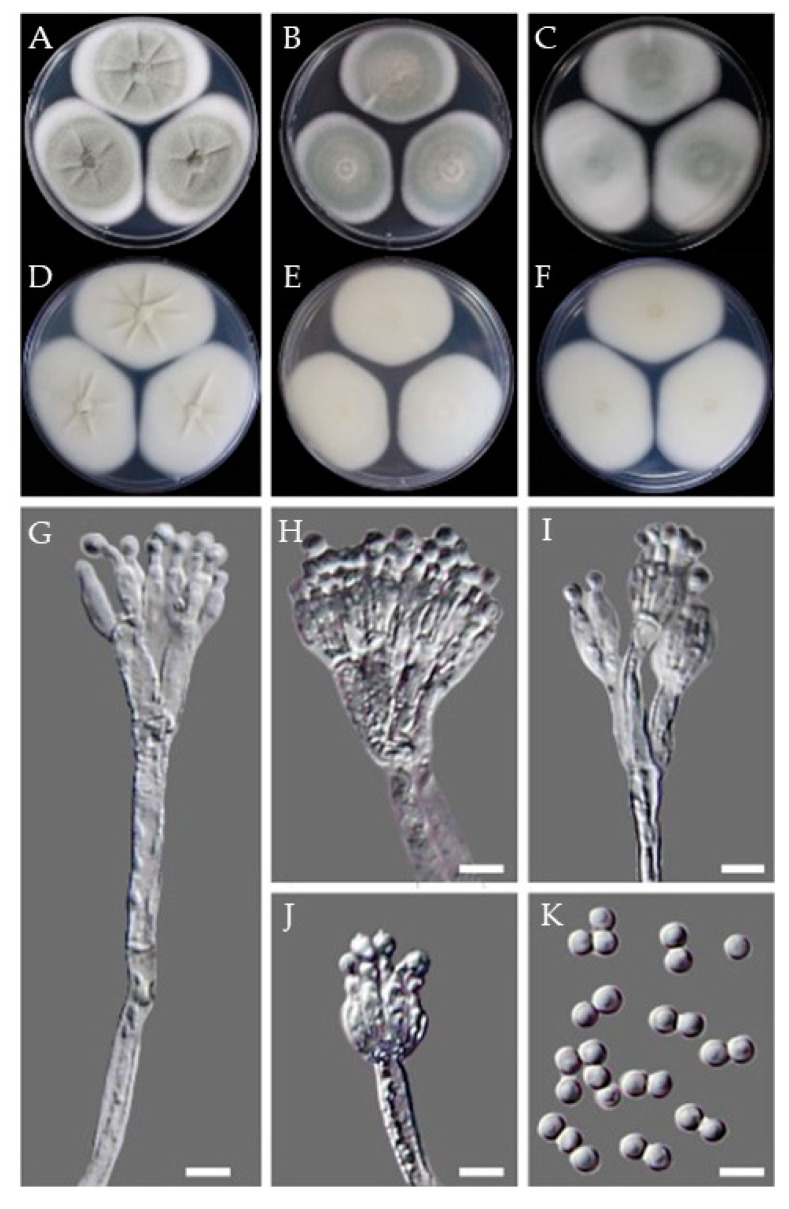
Morphological observations of the isolate CMML21–7. Obverse and reverse colony morphology on CYA (**A**,**D**), MEA (**B**,**E**), and YES (**C**,**F**) after 7 days at 25 °C. Structures and shapes of conidiophores including phialides, metulae, and stipes (**G**–**J**); conidia (**K**). Scale bars, (**G**–**K**) = 5 μm.

Morphology: On CYA at 25 °C: After 7 days colony diameter was 58–60 mm. Colonies off-white to pale primrose, smooth, deep radial furrows extending to sub-margin furrows with cracked ridges. No exudate was observed (Figure 9A,D). On MEA at 25 °C: Colonies white to off-white and reaching 46–50 mm in diameter. A sub-margin was observed, slightly depressed and no obvious conidiogenesis (Figure 9B,E). On YES at 25 °C: Colonies white to off-white and reaching 57–59 mm in diameter, smooth and velvety texture. No obvious conidiogenesis and reverse ochraceous salmon (Figure 9C,F).

Conidiophores are mostly monoverticilliate but occasionally divaricate or biverticilliate; stipes smooth-walled, rarely finely roughened. The sizes of the stipes were 31.0–150.5 × 3.2–3.6 μm; metula 1–2 per stipe, 8.9–12.4 × 1.6–2.6 μm, phialides ampulliform, 5.1–8.7 × 1.7–2.3 μm. Conidia were subglobose to slightly ovoid and smooth-walled, 2.8–3.8 × 2.6–3.6 (Figure 9G–K).

Distribution: The *P. rotoruae* was first described as a new species from in-ground timber in New Zealand [42]. This is the first worldwide report of *P. rotoruae*-caused disease in sweet potatoes.

Note: All four sequences matched well with the fungus *P. rotoruae.* This fungus exhibits relatively fast-growing colonies on CYA at 25 °C but is slower than its relatives (*P. ochrochloron* and *P. svalbardense*). Conidiogenesis generally was not observed but was abundant at 30 °C. Stipes were smooth-walled and rarely roughened; conidia subglobose to slightly ovoid and smooth-walled.

### 3.6. Pathogenicity

All the species were tested for pathogenicity. Each isolate was inoculated on the sweet potato variety ‘Beniharuka’. Pathogenicity tests confirmed their disease-producing ability. After 3 weeks of infection, the result showed that the pathogens infected all the treated sweet potato storage roots and exhibited strong to mild pathogenicity. The virulence of the pathogen and isolates varied at a 5% level of significance (*p* < 0.05). The isolate CMML21–16, which was confirmed as *M. phaseolina*, showed the highest disease severity by producing charcoal rot disease on sweet potato storage roots. The average length and depth of lesions were 112.95 and 42.80 mm, respectively (Table 2).

**Figure 10:**
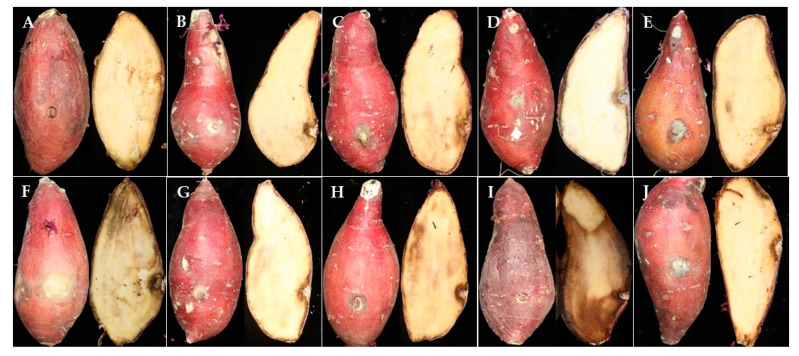
Pathogenicity tests of fungal isolates obtained from this study on the ‘Beniharuka’ variety. (**A**) control, (**B**) *F. oxysporum* CMML21–2, (**C**) *A. wentii* CMML21–4, (**D**) *P. citrinum* CMML21–5, (**E**) *P. rotoruae* CMML21–7, (**F**) *F. ipomoeae* CMML21–8, (**G**) *Mu.* *variicolumellatus* CMML21–12, (**H**) *F. oxysporum* CMML21–13, (**I**) *M. phaseolina* CMML21–15, (**J**) *F. solani* CMML21–17.

The least infected sweet potato was caused by the pathogen *Mu. variicolumellatus* and the average lesion length and depth were 12.65 and 5.03 mm, respectively. Among all the *Fusarium* species, *F. ipomoeae* CMML21–8 caused higher disease infection than the other two species *F. oxysporum* (CMML21–2 and CMML21–13) and *F. solani* (CMML21–17). The newly isolated pathogen *A. wentii* showed the lesion length and depth of 15.32 and 7.80 mm, respectively. In addition, the average lesion length and depth caused by the pathogen *P. rotoruae* were 15.55 and 7.23 mm, respectively, and the species caused higher lesion length and depth than *P. citrinum* (Table 2 and Figure 10).

### 3.7. In Vitro Antifungal Activity

**Figure 11:**
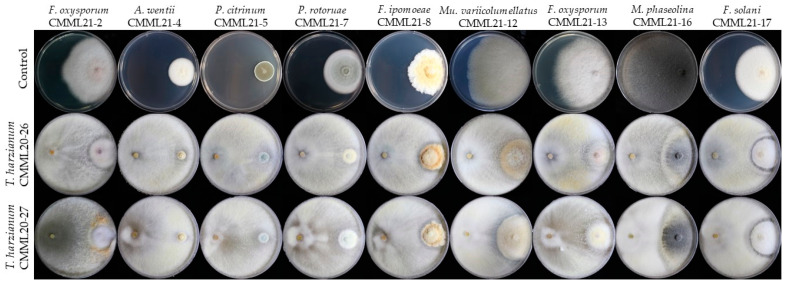
Representative photographs of in vitro dual culture assay for mycelial growth inhibition of 8 different pathogenic species isolated from sweet potato storage roots by *Trichoderma harzianum* strains CMML20–26 and CMML20–27.

Two antagonistic *T. harzianum* strains (CMML20–26 and CMML20–27) were tested against eight different pathogenic species isolated from sweet potato storage roots. Dual culture activity results revealed that the two *T. harzianum* strains showed strong antifungal activity against all tested pathogens. Two *Trichoderma* strains showed strong antagonistic activity against *F. oxysporum* isolates (CMML21–2 and CMML21–13), and the rate of pathogen inhibition was 72.88–81.14%. The newly recovered pathogen in sweet potato *A. wentii* (CMML21–4) was inhibited 73.93–77.04% by the two antagonists. Lower inhibition was observed against *P. citrinum* (CMML21–5) (Table 3). Another new disease-producing pathogen, *P. rotoruae* (CMML21–7), was inhibited by CMML20–26 and CMML20–27 by 69.40 and 58.13%, respectively. The inhibition of *F. ipomoeae* (CMML21–8) by two *T. harziaznum* strains was 56.99–57.06%. The lowest inhibition rate (42.66 to 50.21%) was observed against *Mu. variicolumellatus* (CMML21–12). The pathogen inhibition rate of *M. phaseolina* (CMML21–15) and *F. solani* (CMML21–17) was 75.01–78.22% and 64.94–68.83%, respectively, by these antagonistic fungal strains (Table 3 and Figure 11).

## 4. Discussion

Sweet potato is the eleventh most important food crop globally [10] and is valuable in nutrition content. It contains high levels of carbohydrates and minerals as well as dietary fibers [3,6,37]. Sweet potato is popular in Korea, and the total area of sweet potato production has increased [3]. However, many common fungal diseases are reported worldwide during storage and marketing of this crop, such as Fusarium surface rot, Fusarium storage root rot and end rot, sclerotial circular spot, soft rot, black rot, blue mold, dry rot, and charcoal rot caused by *Fusarium* spp., *F. solani*, *Sclerotium rolfsii*, *Rhizopus* and *Mucor* spp., *Penicillium* spp., *Diaporthe batatas*, and *M. phaseolina*, respectively [3,6,10,15,16,17].

In the present study, surface rot, blue mold, and charcoal rot diseases were commonly observed, and *F. oxysporum*, *F. ipomoeae*, *P. citrinum*, and *M. phaseolina* were frequently isolated. The most frequent pathogen was *F. oxysporum*, which caused surface rot disease. The disease is more damaging than any other storage disease of sweet potato worldwide [3]. In addition, end rot and charcoal rot diseases were found in the study. Several fungal species caused end rot, including *F. solani*, *M. phaseolina*, *Lasiodiplodia theobromae*, and *D. batatas* [20]. *F. solani* was isolated from the end rot disease, and the *M. phaseolina* isolates were isolated from charcoal rot diseases. Charcoal rot of sweet potato is widespread in the tropics [43]; decay usually begins at the end of the storage roots. Initial symptoms are variously shaped and sized pale brown discolorations, and eventually, rotten roots with micro-sclerotia colonize interiorly. The *Mucor* sp. was isolated from rotten sweet potatoes. Multigene molecular phylogeny identified the pathogen as *M. varricolumellatus*, which Wagner named in 2020 [38]. Detailed description and the reference explanation showed that the pathogen belongs to the *Mu. circinelloides* species complex group. *Mu. circinelloides* (Syn. *M. racemosus*) was previously isolated as a pathogen from sweet potato [18].

The pathogenicity test is essential to observe the environmental condition producing the disease and whether the pathogen isolated was virulent or not and to confirm Koch’s postulates. All the pathogens tested for pathogenicity in the present study showed disease symptoms in a different lesion length. The most severe disease was observed on sweet potato storage roots inoculated by the *M. phaseolina* isolate. *Macrophomina* spp. are the most severe and frequent species among fungal isolates recovered from sweet potato stems and storage roots with rot symptoms in Brazil [14]. *M. phaseolina* deserves to be highlighted as it is a widely distributed plant pathogen that can produce microsclerotia that survive in the soil for a long time [14,44]. The pathogenicity tests on storage roots showed disease symptoms, but the disease progression was slow, which is common in storage environments [3,15,20]. Pathogenicity and disease progression depend on many factors, including inoculum density, temperature, and their interactions [45]. Therefore, the degrees of pathogenicity varied with isolates and fungal species.

Two new postharvest disease pathogens, *A. wentii* and *P. rotoruae*, were isolated in the storage roots of sweet potato. *A. wentii* caused black mold or dry rot disease, and *P. rotoruae* caused blue mold disease. The pathogen *A. niger* causes black mold rot in storage roots and has been reported from Bangladesh, China, India, Nigeria, and the USA [46,47]. Mold disease is also a common postharvest disease caused by *Penicillium* spp. including *P. oxalicum* and *P. citrinum* [46,47,48,49]. The present study identified for the first time two pathogens reported to cause disease in sweet potato, and pathogenicity tests confirmed their disease-producing ability. *P. rotoruae* was discovered and described as a new species in New Zealand [42], and the Republic of Korea is the second country where this fungal species has been found.

Postharvest diseases are caused by several factors, such as low pH, moisture content, nutritional composition, improper handling, and management of storage roots during harvest, transport, and marketing. Improper handling causes wounds in storage roots and allows pathogens to enter, thus infecting the storage roots. *Fusarium* spp., *M. phaseolina*, and many other postharvest pathogens enter in such a way [50]. Infested sweet potatoes can cause serious health issues, especially mold pathogens including *Aspergillus* spp. and *Penicillium* spp., which produce mycotoxins that can be lethal to humans [50]. Therefore, proper handling of sweet potato cultivation as well as investigation of possible biocontrol agents and methods are needed to minimize the fungus-mediated infections after harvest. Different species of *Trichoderma* have been used for postharvest biocontrol agents in crops such as papayas, strawberries, tomatoes, apples, pears, and bananas. Three *Trichoderma* species, *T. asperellum*, *T. viride*, and *T. harzianum*, showed strong antagonistic activities against different fungal pathogens [51]. The biocontrol agents *Trichoderma* spp. use various mechanisms against pathogens, including production of antifungal compounds, competition for nutrients, parasitism or inhibition of pathogens, antibiosis, and production of lytic enzymes [51,52]. In this study, we investigated *T. harzianum* strains CMML20–26 and CMML20–27 for dual culture antagonistic activity and observed that these two biocontrol agents could reduce the pathogenic fungal growth up to 85%. A similar antagonistic agent *T. asperellum* was isolated as a pathogen from the sweet potato storage roots, which inhibited the growth of pathogens such as *F. oxysporum*, *F. proliferatum*, *L. theobromae*, and *Rhizopus nigricans* [12]. Therefore, more attention should be given to test alternative biocontrol systems to reduce diseases of sweet potato with *Trichoderma* spp. as well as other biocontrol agents.

## Figures and Tables

**Table 1:** Total number of fungi isolated from postharvest sweet potatoes in Korea during the study in 2021.

Location	Number of Fungi Isolated				
	*Fusarium* spp.	*Penicillium* spp.	*Aspergillus* sp.	*Mucor* sp.	*Macrophomina* sp.
Cheonan-si	12	7	4	–	–
Haenam-gun	24	–	–	1	–
Buan-gun	10	–	–	–	10
No. of fungi	46	7	4	1	10
Isolation (%)	(67.6%)	(10.3%)	(5.9%)	(1.5%)	(14.7%)
Total	68				

**Table 2:** The pathogenicity of the pathogenic species obtained in the present study in a sweet potato variety (Beniharuka) 3 weeks after surface wound inoculation.

Fungal Isolates	Disease Length (mm)	Disease Depth (mm)
*Fusarium oxysporum* CMML21–2	13.52 ± 0.95 c	6.67 ± 1.92 b
*Aspergillus wentii* CMML21–4	15.32 ± 0.61 c	7.80 ± 0.95 b
*Penicillium citrinum* CMML21–5	12.96 ± 1.02 c	6.20 ± 0.66 b
*Penicillium rotoruae* CMML21–7	15.55 ± 0.32 c	7.23 ± 0.39 b
*Fusarium ipomoeae* CMML21–8	23.47 ± 2.09 b	9.30 ± 1.11 b
*Mucor variicolumellatus* CMML21–12	12.65 ± 0.58 c	5.03 ± 1.47 b
*Fusarium oxysporum* CMML21–13	16.85 ± 2.22 bc	9.27 ± 1.13 b
*Macrophomina phaseolina* CMML21–16	112.95 ± 2.25 a	42.80 ± 0.30 a
*Fusarium solani* CMML21–17	14.0 6 ± 2.03 c	6.90 ± 1.15 b

Note. Data of length on surface and depth of the disease spot in the table are the mean ± SE. Different lowercase letters after data indicate significant differences among isolates (*p* < 0.05). Values with the same letters are not significantly different.

**Table 3:** Antifungal activity of *Trichoderma harzianum* strains CMML20–26 and CMML20–27 against all the pathogenic species recovered in the present study.

Fungal Isolates	Inhibition Rate (%)	
	CMML20–26	CMML20–27
*Fusarium oxysporum* CMML21–2	81.14 ± 3.07 a	72.05 ± 0.92 ab
*Aspergillus wentii* CMML21–4	77.04 ± 0.69 abc	73.93 ± 2.50 a
*Penicillium citrinum* CMML21–5	53.87 ± 2.04 d	53.87 ± 0.48 de
*Penicillium rotoruae* CMML21–7	69.40 ± 0.51 bc	58.13 ± 1.35 cd
*Fusarium ipomoea* CMML21–8	56.99 ± 3.44 d	57.06 ± 1.60 cd
*Mucorvariicolumellatus* CMML21–12	50.21 ± 1.27 d	42.66 ± 1.28 e
*Fusarium oxysporum* CMML21–13	75.29 ± 0.49 abc	72.88 ± 2.99 ab
*Macrophominaphaseolina* CMML21–16	78.22 ± 0.91 ab	75.01 ± 2.50 a
*Fusarium solani* CMML21–17	68.83 ± 0.89 c	64.94 ± 0.65 bc

Note: Data of inhibition rate in the table are the mean ±SE. Different lowercase letters after data indicate significant differences among strains (*p* < 0.05). Values with the same letters are not significantly different.

## Data Availability

GenBank accession numbers for the microorganism’s sequence will be available soon and accession numbers are OK104035–OK104051 for ITS, OK104467–OK104475 for EF1, OK104452–OK104456 for BT, OK104462–OK104466 for RPB2, OK175702 for LSU, and OK175704 for SSU.

## Supplementary Materials

The following are available online at https://www.mdpi.com/article/10.3390/jof7110927/s1, Table S1, List of fungal isolates from postharvest sweet potatoes in Korea and primers were used for identification of the isolates in this study. Table S2, List of fungal species, strain number, and GenBank accession numbers of sequences used in this study.
