# Supplementary material for: Fungi Associated with Postharvest Diseases of Sweet Potato Storage Roots and In Vitro Antagonistic Assay of Trichoderma harzianum against the Diseases
Source: J Fungi (Basel). 2021 Oct 31;7(11):927. doi: 10.3390/jof7110927 (PMC8625119; doi:10.3390/jof7110927)
Supplement: Supplementary file 1 [file jof-07-00927-s001.zip › jof-1413221-supplementary.pdf]

Supplementary Table S1. List of fungal isolates from postharvest sweet potatoes in Korea and primers were used for identification of the isolates in this study.

Isolates	Name of fungi	Primer used	Location
CMML21–1[1]	*Fusarium oxysporum*	ITS/EF[2]	Cheonan–si
CMML21–2	*Fusarium oxysporum*	ITS/EF	Cheonan–si
CMML21–3	*Aspergillus wentii*	ITS/BT/RPB2/CAL	Cheonan–si
CMML21–4	*Aspergillus wentii*	ITS/BT/RPB2/CAL	Cheonan–si
CMML21–5	*Penicillium citrinum*	ITS/BT/RPB2/CAL	Cheonan–si
CMML21–6	*Penicillium citrinum*	ITS/BT/RPB2/CAL	Cheonan–si
CMML21–7	*Penicillium rotoruae*	ITS/BT/RPB2/CAL	Cheonan–si
CMML21–8	*Fusarium ipomoeae*	ITS/EF	Haenam–gun
CMML21–9	*Fusarium ipomoeae*	ITS/EF	Haenam–gun
CMML21–10	*Fusarium oxysporum*	ITS/EF	Haenam–gun
CMML21–11	*Fusarium oxysporum*	ITS/EF	Haenam–gun
CMML21–12	*Mucor variicolumellatus*	ITS/SSU/LSU	Haenam–gun
CMML21–13	*Fusarium oxysporum*	ITS/EF	Buan–gun
CMML21–14	*Fusarium oxysporum*	ITS/EF	Buan–gun
CMML21–15	*Macrophomina phaseolina*	ITS/EF	Buan–gun
CMML21–16	*Macrophomina phaseolina*	ITS/EF	Buan–gun
CMML21–17	*Fusarium solani*	ITS/EF	Cheonan–si

[1]CMML indicates Chonnam National University Molecular Microbiology Laboratory and fungi isolated in the present study; [2]primers used were internal transcribed spacer (ITS), elongation factor 1-alpha (EF1-α), β-tubulin (BT), RNA polymerase II second largest subunit (RPB2), Small subunit (SSU), and Large Subunit (LSU).

Supplementary Table S2. List of fungal species, strain number, and GenBank accession numbers of sequences used in this study.

Species	Strain no.	ITS	BT	CAM	EF1	RPB2	SSU	LSU
Aspergillus arxii	CBS 525.83	MH861638.1	MN969365.1	MN969223.1	-	-	-	-
Aspergillus caninus	CBS:128032	KY980618	KY980546.1	MN969225.1	-	-	-	-
Aspergillus cervinus	CBS 196.64	MH858414.1	FJ491634.1	FJ491595.1	-	-	-	-
Aspergillus glaucus	NRRL 116	EF652052	EF651887	LC589321.1	-	JN121527	-	-
Aspergillus inflatus	CBS 682.70	MH859900.1	FJ531008.1	FJ531090.1	-	-	-	-
Aspergillus insolitus	CBS 384.61	KY980622.1	KY980550.1	KY980586.1	-	-	-	-
Aspergillus pulvinus	CBS 578.65	FJ531048.1	FJ531013.1	FJ531086.1	-	-	-	-
Aspergillus sclerotialis	CBS 366.77	MH861076.1	KY980579.1	KY980615.1	-	-	-	-
Aspergillus wentii	**CMML21–3**	**OK104044**	**OK104452**	**OK104457**	**-**	**OK104462**	-	-
Aspergillus wentii	**CMML21–4**	**OK104045**	**OK104453**	**OK104458**	**-**	**OK104463**	-	-
Aspergillus wentii	CBS 104.07	FJ531076.1	FJ531034.1	FJ531116.1	-	-	-	-
Aspergillus wentii	CBS 118.34	MH855467	FJ531037.1	FJ531113.1	-	-	-	-
Botryosphaeria dothidea	CMW8000	AY236949	-	-	AY236898	-	-	-
Fusarium asiaticum	NRRL 26156	NR_121320	-	-	AF212452	-	-	-
Fusarium bactridioides	CBS 100057	MH862686	-	-	MN533993	-	-	-
Fusarium begoniae	CBS:452.97	MH862660	-	-	MN533994	-	-	-
Fusarium biseptatum	CBS 110311	NR_137706	-	-	EU926319	-	-	-
Fusarium cerealis	MAFF 241212	AB820717	-	-	AB820701	-	-	-
Fusarium circinatum	CBS:405.97	MH862654	-	-	KM231943	-	-	-
Fusarium denticulatum	CBS 407.97	NR_138359	-	-	MN534000	-	-	-
Fusarium equiseti	NRRL 26419	NR_121457	-	-	GQ505599	-	-	-
Fusarium fujikuroi	CBS 221.76	MW827608	-	-	MN534010	-	-	-
Fusarium graminearum	CBS 130917	JX162342	-	-	JX118950	-	-	-
Fusarium incarnatum	CBS 133024	KF255449	-	-	KF255493	-	-	-
Fusarium ipomoeae	**CMML21–8**	**OK104041**	**-**	**-**	**OK104473**	**-**	**-**	**-**
Fusarium ipomoeae	**CMML 21–9**	**OK104042**	**-**	**-**	**OK104474**	**-**	**-**	**-**
Fusarium ipomoeae	CQ1099	MK280853	-	-	MK289573	-	-	-

Fusarium ipomoeae	LC12163	MK280790	-	-	MK289597	-	-	-
Fusarium oxysporum	**CMML 21–1**	**OK104035**	**-**	**-**	**OK104467**	**-**	-	-
Fusarium oxysporum	**CMML 21–2**	**OK104036**	**-**	**-**	**OK104468**	**-**	-	-
Fusarium oxysporum	**CMML 21–10**	**OK104037**	**-**	**-**	**OK104469**	**-**	-	-
Fusarium oxysporum	**CMML 21–11**	**OK104038**	**-**	**-**	**OK104470**	**-**	-	-
Fusarium oxysporum	**CMML 21–13**	**OK104039**	**-**	**-**	**OK104471**	**-**	-	-
Fusarium oxysporum	**CMML 21–14**	**OK104040**	**-**	**-**	**OK104472**	**-**	-	-
Fusarium oxysporum	NRRL:34118	MK806586	-	-	MK818423	-	-	-
Fusarium oxysporum	NRRL:38352	MK806587	-	-	MK818418	-	-	-
Fusarium oxysporum	FS11476a	MN416203	-	-	MN417194	-	-	-
Fusarium oxysporum	CBS 129.24	MH854774	-	-	MH484955	-	-	-
Fusarium oxysporum	SPL15020	KY508368	-	-	KY508356	-	-	-
Fusarium oxysporum	SPL16048	KY508357	-	-	KY508345	-	-	-
Fusarium polyphialidicum	CBS 676.94	KR071663	-	-	KU604469	-	-	-
Fusarium solani	**CMML 21–17**	**OK104043**	**-**	**-**	**OK104475**	-	-	-
Fusarium solani	SPL16128	KY796230	-	-	KY796236	-	-	-
Fusarium solani	SPL16098	KY796225	-	-	KY796231	-	-	-
Fusarium solani	CBS 102429	KM231808	-	-	KM231936	-	-	-
Fusarium temperatum	EFA313A	KC179826	-	-	KC179824	-	-	-
Macrophomina euphorbiicola	CMM 4045	KU058928	-	-	KU058898	-	-	-
Macrophomina euphorbiicola	CMM 4134	KU058936	-	-	KU058936	-	-	-
Macrophomina phaseolina	**CMML 21–15**	**OK104049**	**-**	**-**	**OK104476**	-	-	-
Macrophomina phaseolina	**CMML 21–16**	**OK104050**	**-**	**-**	**OK104477**	-	-	-
Macrophomina phaseolina	CBS 227.33	KF951627	-	-	KF952000	-	-	-
Macrophomina phaseolina	CBS 162.25	KF531826	-	-	KF951996	-	-	-
Macrophomina phaseolina	CPMM	KT261801	-	-	KT261797	-	-	-
Macrophomina pseudophaseolina	CPC 21524	KF951799	-	-	KF952161	-	-	-
Macrophomina pseudophaseolina	CPC 21501	KF951796	-	-	KF952158	-	-	-
Macrophomina vaccinii	CGMCC3.19510	MK687457	-	-	MK687433	-	-	-
Macrophomina vaccinii	CGMCC3.19509	MK687456	-	-	MK687432	-	-	-

Mucor amethystinus	CBS 526.68	JN206015	-	-	-	-	NG_076771	JN206426
Mucor circinelloides	CBS 195.68	JN205961	-	-	-	-	NG_065588	NG_055735
Mucor circinelloides	CBS 384.95	MH862534	-	-	-	-	HM623317	MH874168
Mucor griseocyanus	CBS 116.08	MH854579	-	-	-	-	MT523933	JN206421
Mucor lusitanicus	CBS 276.49	JN205984	-	-	-	-	MT523932	MT523850
Mucor lusitanicus	CBS 108.17	JN205980	-	-	-	-	JF723683	MH866177
Mucor pseudocircinelloides	CBS 541.78	JN206013	-	-	-	-	NG_076765	NG_073591
Mucor racemosus f. *racemosus*	CBS 260.68	MH859131	-	-	-	-	MT523919	MH870843
Mucor ramosissimus	CBS 135.65	JN205932	-	-	-	-	NG_076769	HM849678
Mucor variicolumellatas	**CMML 21–12**	**OK104051**	-	-	-	-	**OK175704**	**OK175702**
Mucor variicolumellatus	CBS 236.35	JN205979	-	-	-	-	NG_076770	JN206422
Penicillium camponotum	NN072443	KY495026	KY495135	KY494966	-	KY495077	-	-
Penicillium citrinum	**CMML 21–5**	**OK104046**	**OK104454**	**OK104459**	**-**	**OK104464**	-	-
Penicillium citrinum	**CMML 21–6**	**OK104047**	**OK104455**	**OK104460**	**-**	**OK104465**	-	-
Penicillium citrinum	DSM 1997	LKUP01000077.1: 4646-5125	LKUP01000476.1: 37082-37624	LKUP01000764.1: 27766-28220	-	LKUP01000752.1: 4836-5927	-	-
Penicillium citrinum	JCM 22607	LC228681.1	BCKA01000001.1: 1251311-1251853	BCKA01000007.1: 1371782-1372236	-	BCKA01000004.1: 2110131-2111222	-	-
Penicillium citrinum	CBS 139.45	MH856132	GU944545	KM089072	-	JF417416	-	-
Penicillium gorlenkoanum	CBS 408.69	GU944581	GU944520	MN969259	-	JN606601	-	-
Penicillium ochrochloron	DTO 189-A6	KC346347	KC346324.1	KC346341.1	-	KC346318.1	-	-
Penicillium ochrochloron	CBS357.48	GU981604	GU981672	KC346340	-	KF296445	-	-
Penicillium piscarium	CBS362.48	GU981600	GU981668	MN969288	-	KF296451	-	-
Penicillium rotoruae	**CMML 21–7**	**OK104048**	**OK104456**	**OK104461**	**-**	**OK104466**	-	-
Penicillium rotoruae	CBS 145838	MN315103.1	MN315104.1	MN315102.1	-	MT240842.1	-	-
Penicillium sizovae	CBS 413.69	MH859338.1	GU944535	MN969298.1	-	JN606603.1	-	-
Penicillium soliforme	NN072399	KY495022	KY495131	KY494962	-	KY495074	-	-
Penicillium svalbardense	CBS122416	NR_111508	GU981669	KC346338	-	KC346315	-	-
Penicillium vasconiae	CBS339.79	MH861218	GU981653	MN969309	-	MN969144	-	-
Talaromyces flavus	CBS 310.38	JN899360.1	JX494302.1	FJ530982.1	-	-	-	-

Note: Sequences and accession numbers obtained in the present study are in bold.
